# Refractory hypotension during general anesthesia despite preoperative discontinuation of an angiotensin receptor blocker

**DOI:** 10.12688/f1000research.2-12.v1

**Published:** 2013-01-14

**Authors:** Raha Nabbi, Harvey J Woehlck, Matthias L Riess

**Affiliations:** 1 Department of Anesthesiology, Medical College of Wisconsin, Milwaukee, WI, 53226, USA; 2 Department of Physiology, Medical College of Wisconsin, Milwaukee, WI, 53226, USA; 3 Clement J. Zablocki VA Medical Center, Milwaukee, WI, 53295, USA

## Abstract

Due to their beneficial reduction in morbidity and mortality angiotensin receptor blockers (ARBs) have become increasingly popular to treat hypertension. However, similar to angiotensin converting enzyme inhibitors, they can lead to severe hypotension in conjunction with general anesthesia and thus have been recommended to be withheld in the morning of surgery. Here, we present a 51 year old female who developed severe refractory hypotension after induction of general anesthesia, although she had discontinued her medication 24 hours preoperatively as instructed. Therefore, halting ARBs for more than 24 hours before surgery may be necessary. Heightened awareness of this potential interaction and recognizing the need to treat with vasopressin is required when ARB-induced hypotension occurs.

## Introduction

Angiotensin II receptor blockers (ARBs) represent a newer class of effective and well tolerated antihypertensive agents ^1^. Several clinical studies have indicated the beneficial effects of ARBs in hypertensive patients such as reduction of left ventricular hypertrophy, decrease in ventricular arrhythmias, and improved diastolic function ^1^. Inhibitors of the renin-angiotensin system (RAS), either angiotensin converting enzyme (ACE) inhibitors or ARBs, mediate vasodilation and consequently decrease blood-pressure by different mechanisms ^1^. ARBs specifically inhibit angiotensin II from binding to its receptor, the Angiotensin-1 (AT _1_) receptor on vascular smooth muscle cells. This blockade results in increased angiotensin II and normal bradykinin plasma levels. ARBs were developed to overcome several deficiencies of ACE inhibitors, which, by comparison, lead to decreased angiotensin II, but increased bradykinin levels. Hence, the key advantage of ARBs over ACE inhibitors is their lack of adverse effects related to bradykinin potentiation. ARBs have been shown to reduce morbidity and mortality associated with hypertension, and therefore, it is not surprising that an increasing number of patients scheduled for surgery are chronically treated with ARBs ^2^. However, RAS blockade increases the risk of severe hypotension during and after anesthetic induction. ACE-inhibitors are well known for inducing severe circulatory side effects during anesthesia, which led to the general recommendation to withhold the drug on the day of surgery ^3^. Similarly, refractory hypotension has been described after induction of general anesthesia in patients chronically treated with ARBs who took their medication in the morning of surgery ^3^. Here, we describe a case of severe hypotension under general anesthesia refractory to conventional treatment, *despite* preoperative discontinuation of an ARB for 24 hours.

## Case report

A 51 year old female (60 kg) whose hypertension was well controlled with Diovan-HCT (valsartan/hydrochlorothiazide 160/12.5 mg) was scheduled for a T2-10 posterior thoracic fusion. As instructed, she had last taken Diovan-HCT 24 hours prior to surgery. Her preoperative exam was unremarkable. Non-invasive blood pressure measurement, 5-lead ECG with ST segment analysis, heart rate, and pulseoximetry were continuously monitored. Her baseline vital signs were stable with a blood pressure of 136/94 mmHg, heart rate of 86 bpm and SpO _2_ 99% on room air. After induction of anesthesia with 150 mg propofol, 100 mcg fentanyl, and 60 mg rocuronium and an uneventful intubation, her blood pressure decreased and remained 65–75/35–45 mmHg for 45 min with a HR of 75–85 bpm despite rapid administration of 1500 cc Lactate Ringer, repeated 100 mcg phenylephrine boluses followed by a phenylephrine drip and repeated boluses of vasopressin (cumulative dose of 20 units within 25 min). Sevoflurane for anesthesia maintenance was kept low at 0.8 Vol%. Due to her refractory hypotension, the decision was made to postpone the patient’s elective surgery and awaken her. Upon emergence, her blood pressure recovered to 115/65 mmHg with a heart rate of 90 bpm, and she was extubated successfully after neuromuscular blocker reversal. The patient did not suffer any neurologic sequelae. Her ARB was withheld postoperatively and she was successfully anesthetized with the same drugs and operated on five days later without significant hypotension.

## Discussion

Valsartan is a potent, highly selective antagonist of the angiotensin II at the AT _1_ receptor leading to vasodilatation and an anesthetic-induced reduction in pre- and afterload. Vasodilation may also be afforded in part by upregulated angiotensin II activating AT _2_-receptors which causes vascular relaxation ^4^ and reduces peripheral vascular resistance usually without a rise in heart rate. The efficacy, tolerability and safety of valsartan have been demonstrated in large-scale studies on patients with hypertension, heart failure and post-myocardial infarction ^5^. Valsartan’s mechanism of action is to displace angiotensin II from the AT _1_ receptor, thereby antagonizing AT _1_-induced vasoconstriction, aldosterone, catecholamine and arginine-vasopressin release, water intake, and hypertrophic responses. All of this results in a more efficient blockade of angiotensin II’s cardiovascular effects and in fewer side effects than ACE inhibitors. In addition, most ARBs control blood pressure for 24 hrs after a single dose.

ARBs are non-peptide compounds, and variations in molecular structure result in different binding affinities to their receptors and different pharmacokinetic profiles ^1^. In comparison to other ARBs, valsartan’s plasma elimination half life is of an intermediate duration (5–10 hrs) ^6^. It is eliminated mainly by non-renal routes. However, protein binding greatly affects its biological half life in the body. Valsartan is highly bound to plasma proteins (94–97%), and these may act as a reservoir or depot from which the drug is slowly released and therefore exhibits a longer lasting effect on the vasculature ^6^. As the unbound drug is metabolized and excreted from the body, some of the bound fraction is released in order to maintain equilibrium. In fact, our case demonstrates impressively that valsartan’s biological half life is far outlived by its physiological effects in the human body and can consequently result in severe hypotension despite its prior discontinuation in cases when RAS activation is needed to maintain hemodynamic stability, as for instance during anesthesia. Indeed, during general anesthesia, maintenance of normotension becomes RAS-dependent ^7^ and a pronounced anesthetic-induced hypotension may be prevented or at least attenuated by angiotensin II-mediated AT _1_ receptor activation. Conversely, by blocking RAS, systemic blood pressures can decrease markedly during general anesthesia ^4^.

In addition, chronic AT _1_ blockade also reduces the vasoconstrictor response to α _1_ receptors activated by norepinephrine, which explains why ARB-induced hypotension can be so resistant to phenylephrine, ephedrine and norepinephrine ^2, 8^ as observed in our patient. The lack of response to repeated phenylephrine boluses and a continuous infusion, fluids and a decrease of the volatile anesthetic urgently required a different approach, and we administered vasopressin in repeated boluses. Clinical studies have shown significant vasoconstrictor effects of vasopressin and increased cardiac filling during echocardiographic measurements ^2^. Vasopressin or its synthetic analogues can restore the sympathetic response and may be useful pressors in cases of refractory hypotension during anaphylaxis ^9^ and septic shock ^10^ as well as in patients on RAS inhibitors, although norepinephrine has been reported to have a more favorable effect on splanchnic perfusion and oxygen delivery ^11^.

We conclude that, similar to ACE inhibitors ^3^, ARBs also need to be withheld for 24 hours prior to elective surgery, and – as our case illustrates – possibly longer to elude unnecessary morbidity and mortality associated with refractory hypotension following induction of general anesthesia in patients on chronic ARB treatment. Heightened awareness of this perilous interaction and recognizing the need to treat with an adequate dose of vasopressin or norepinephrine is required, when ARB-induced hypotension is encountered.

## Consent

This case report is in accordance with institutional IRB-guidelines. We have been unable to obtain explicit written consent from the patient.
