# Supplementary material for: Refractory hypotension during general anesthesia despite preoperative discontinuation of an angiotensin receptor blocker
Source: F1000Res. 2013 Jan 14;2:12. [Version 1] doi: 10.12688/f1000research.2-12.v1 (PMC3752674; doi:10.12688/f1000research.2-12.v1)
Supplement: Peer review history

# Peer review: Referee response for version 1

**Published:** 2013-01-22

**Authors:** Yukio Hayashi

**Affiliations:** Department of Anesthesiology, Osaka University Medical School, Osaka, Japan

This report may give us several suggestions and it may be helpful to consider the anesthetic management of similar patients.

I have read this submission. I believe that I have an appropriate level of expertise to confirm that it is of an acceptable scientific standard.

# Peer review: Referee response for version 1

**Published:** 2013-01-17

**Authors:** Sumio Hoka

**Affiliations:** Department of Anesthesiology and Critical Care Medicine, Kyushu University, Kanagawa, Japan

I have read this submission. I believe that I have an appropriate level of expertise to confirm that it is of an acceptable scientific standard.
